# Challenges and Opportunities in Quantifying Bioactive Compounds in Human Breastmilk

**DOI:** 10.3390/biom15030325

**Published:** 2025-02-24

**Authors:** Amna Ghith, Reza Maleki, Luke E. Grzeskowiak, Lisa H. Amir, Wendy V. Ingman

**Affiliations:** 1 Discipline of Surgical Specialties, Adelaide Medical School, University of Adelaide, The Queen Elizabeth Hospital, Woodville South, SA 5011, Australia; amna.ghith@adelaide.edu.au (A.G.); reza.maleki@adelaide.edu.au (R.M.); 2 Robinson Research Institute, University of Adelaide, Adelaide, SA 5006, Australia; 3 College of Medicine and Public Health, Flinders Health and Medical Research Institute, Flinders University, Bedford Park, SA 5042, Australia; luke.grzeskowiak@flinders.edu.au; 4 SAHMRI Women and Kids, South Australian Health and Medical Research Institute, Women’s and Children’s Hospital, North Adelaide, SA 5006, Australia; 5 Judith Lumley Centre, School of Nursing and Midwifery, La Trobe University, Bundoora, VIC 3086, Australia; l.amir@latrobe.edu.au; 6 Breastfeeding Service, The Royal Women’s Hospital, Parkville, VIC 3050, Australia

**Keywords:** breastmilk, lactation, bioactive compounds, biomarker discovery, quantification, validation

## Abstract

Breastmilk is a complex biological fluid containing over a thousand bioactive proteins, lipids, cells and small molecules that provide nutrition and immunological protection for infants and children. The composition of breastmilk is unique to each individual and can also vary within individuals according to breastfeeding duration, maternal health, time of day, and other factors. As such, the composition of breastmilk can be considered a “fingerprint” that could be interrogated to identify biomarkers of breast health and disease. However, accurate quantification of bioactive components in breastmilk remains a significant challenge. Approaches such as immunoassays and mass spectrometry have been largely applied to study blood or other biological fluids and require validation and optimisation before these techniques can be used to accurately quantify bioactive compounds in breastmilk. Development of protocols specific to breastmilk should be carried out with high precision, confidence, and sensitivity. This review explores the challenges and opportunities associated with different techniques for quantification of breastmilk bioactive components.

## 1. Introduction

Healthy infant development requires the production of sufficient volumes of breastmilk during lactation with the right balance of both macro and micronutrients [1]. Breastmilk is a complex biological fluid that contains over a thousand bioactive proteins, lipids, cells, carbohydrates, hormones and immune factors. The concentrations of these bioactive components can change dramatically over a single feed and over time to meet the growing infant’s needs [2]. There are other factors that affect the composition of breastmilk such as the health of the mother and child, maternal stress, and diet [2]. The bioactive components of breastmilk are unique for each woman and can be considered a “fingerprint” that may be indicative of healthy lactation or the risk of disease states such as mastitis or breast cancer.

**Figure 1:**
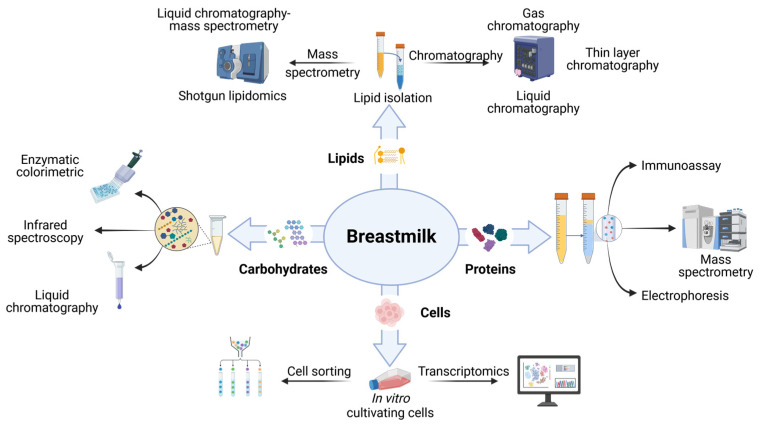
Summary of the methods used to quantify proteins, lipids, carbohydrates and cells in breastmilk.

To investigate the role of different breastmilk components on functional lactation and breast health, we must be able to accurately and reliably quantify these compounds. Many experimental approaches exist to measure proteins, lipids, carbohydrates and cells in breastmilk, including mass spectrometry, immunoassays, liquid chromatography, lipidomics and transcriptomics (Figure 1). However, these approaches rely on protocols used largely to investigate other biological fluids such as blood. Adoption of these techniques to breastmilk poses challenges which need to be considered. The overall biological composition of a fluid is called the “matrix”, and this can have a dramatic impact on how the fluid should be stored, transported, and analysed. There is a high variation between the matrices of different biological fluids such as blood and breastmilk [3]. Therefore, techniques developed to measure components in blood require thorough optimisation and validation before use in breastmilk.

Despite numerous studies that have investigated the bioactive components of breastmilk using immunoassay and mass spectrometry approaches, quantification remains a significant challenge requiring optimisation in sample pre-processing and assay validation. This review provides an in-depth exploration of the challenges and opportunities involved in quantifying bioactive components of breastmilk.

## 2. Bioactive Components of Breastmilk

Breastmilk is made up of approximately 88% water, with the remaining volume consisting of solid macro- and micronutrients as well as non-nutrient compounds. Macronutrients include carbohydrates at about 7% (67–78 g/L), proteins at approximately 1% (9–12 g/L), and lipids at around 3.8% (32–36 g/L) [2,4,5,6,7]. Proteins and peptides, including caseins, whey proteins, immunoglobulins, and lysozyme, and specific amino acids, such as taurine and polyamines, provide antimicrobial, immunomodulatory, and antioxidant activity [8,9,10,11]. The fat layer in breastmilk consists of different lipid species, including phospholipids that promote brain [8,9,10], gut [12] and mucosal development, and immune function [13,14,15]. Additionally, the lipid component contains long-chain polyunsaturated fatty acids, such as arachidonic acid and docosahexaenoic acid which are known to have a substantial impact on brain development and cognitive and immune function [16,17]. Carbohydrates, including lactose and human milk oligosaccharides (HMOs), serve as energy sources, prebiotics, and immune protectants [13,14,15].

Non-nutrient roles of HMOs include promoting infant gut maturity and gut health [18]. HMOs can be categorised into three main groups: neutral fucosylated HMOs, which constitute 35–50% of the total HMOs, acidic sialylated HMOs, making up 12–14%, and neutral non-fucosylated HMOs, which account for 42–55% of total HMOs [19,20]. They act as prebiotics by enriching beneficial intestinal bacteria and shaping the gut microbiota. In addition, HMOs exhibit antimicrobial, antiviral, and anti-inflammatory effects [18,21]. They are also known to have a role in maintaining immune homeostasis as they can interact with different immune cells such as dendritic cells, T cells, and B cells, and influence the expression of both pro- and anti-inflammatory cytokines [14,22]. Due to their diverse biological functions and their significant therapeutic potential, they are being explored to combat a wide variety of diseases including allergies, type 1 diabetes mellitus, rheumatoid arthritis, multiple sclerosis, necrotizing enterocolitis, and inflammatory bowel disease, although clinical research is needed to evaluate their effectiveness [23,24,25].

Micronutrients such as vitamins (A, D, E, K, and B-complex) and minerals (calcium, phosphorus, and zinc) support metabolic processes as well as brain and skeletal muscle development [26,27,28]. Enzymes such as lipase and amylase aid digestion [29], while hormones and growth factors such as epidermal growth factor and insulin-like growth factor enhance tissue growth and intestinal maturation and healing [2].

Other proteins in breastmilk such as antibodies, cytokines and lactoferrin play a crucial role in supporting the newborn’s developing immune system [30,31]. Cytokines are small bioactive proteins which play a crucial role in regulating the body’s immune response and they can also act as biomarkers for inflammation [10]. Lactoferrin is an iron-binding glycoprotein that not only enhances immune function but also exhibits antibacterial, antiviral, and antifungal activities [32,33,34]. Moreover, breastmilk contains microRNAs which are small non-coding RNAs. The most abundant microRNA in breastmilk is the microR-148 which is hypothesised to act to fine tune innate immune responses [35]. Carr et al. have also suggested that the microRNA microR-21 can regulate the innate immune system [30]. Finally, the presence of antioxidants in breastmilk may contribute to protection against oxidative stress [36,37].

## 3. Opportunities for Breastmilk Research to Advance Maternal and Infant Health

The variability between one mother’s breastmilk and another is what makes it a unique “fingerprint” tailored specifically to meet the baby’s needs, which can also provide insight into both mother’s and baby’s health. Investigation of the variability in bioactive components of breastmilk provides opportunities to better understand changes in infant needs over time, determinants of infant health, and also the risk of disease states in the mother including mastitis and breast cancer.

Breastmilk transitions from colostrum in the first days postpartum to mature breastmilk at four to six weeks postpartum, with distinct differences in nutrient profiles [38,39]. Colostrum is nutrient-dense breastmilk with high concentrations of antibodies and proteins, and is lower in lactose compared to mature breastmilk. The bioactive components found in colostrum can vary between term and preterm infants [40]. For example, the levels of lactose and short- and medium-chain fatty acids increase, while the concentration of HMOs decreases when progressing from colostrum to mature breastmilk in preterm and full-term infants [41,42,43].

Breastmilk composition also varies throughout the day. Evening breastmilk is enriched with components that help the baby to relax and sleep, whereas daytime breastmilk contains different elements that stimulate activity [44,45]. Cortisol is a hormone known to promote alertness and is three times higher in morning milk compared to evening milk [46]. Meanwhile, melatonin, which has both hypnotic and relaxing effects, is almost undetectable in daytime milk but increases in the evening, peaking around midnight, to support better nocturnal sleep [47,48]. In addition, higher levels of nucleotides in night milk act as natural sleep inducers [44] in contrast to daytime milk, which contains more activity-promoting amino acids [45].

Breastmilk can also change in response to the baby or mother’s illness, with an increase in immune cell levels to aid recovery [49,50,51]. Breastmilk composition continues to change as the infant develops. After the baby’s first year, the fat and energy content of breastmilk increases to meet their growing needs [52]. There are also many other factors that contribute to the high variability in breastmilk composition such as maternal diet, genetics, ethnicity, and environmental factors [53,54,55,56].

Investigating how human breastmilk components influence anthropometry and child health can help identify early risk factors and predictors of obesity, offering valuable insights into promoting healthy growth and development [57]. In addition, breastmilk components serve as a valuable resource for exploring aetiology and biomarker discovery for breast inflammatory diseases such as lactational mastitis [58,59].

As breastmilk originates in the alveoli and travels through the breast ducts, it is considered a window into the breast microenvironment and the risk of breast cancer [60,61,62,63]. It has potential as a diagnostic biological fluid for non-invasive biomarker discovery for breast cancer [64,65,66] and may have use in cases where traditional imaging (e.g., mammogram) is less effective, such as in women with dense breast tissue [60,67,68,69].

## 4. Challenges in Quantification of Breastmilk Bioactive Components

There are many factors that can affect the quantification of bioactive compounds in breastmilk, including pre-analytical and analytical challenges. Pre-analytical processing includes critical stages such as collection, storage, transport, and sample preparation, all of which require careful standardisation. Improper collection techniques, inadequate storage conditions, and suboptimal handling during transport can significantly alter the total protein concentration, thereby affecting the accuracy of analytical protocols and the reliability of subsequent methodological tests [70,71]. An important analytical challenge is the matrix effect, where most quantification methods have been developed and validated in blood or other biological fluids, which have a different matrix from breastmilk.

Breastmilk sample collection timing and frequency need to be considered during the study design [2,72,73,74]. For optimal comparability, the timing of collection with respect to infant age should be standardised between participants [73,75,76,77,78]. Another important consideration is ensuring the breast used for collection throughout the study is the same, especially if collection is performed longitudinally across different lactation stages [77,78,79]. The volume of breastmilk produced is impacted by feeding; therefore, it is recommended to avoid collecting breastmilk within two hours of the last feeding. This will ensure an adequate volume of breastmilk is produced [75,78,80].

The use of aseptic or non-aseptic methods of collecting breastmilk can also affect analysis [81], and this should be documented in the protocol. Expressed breastmilk may be contaminated by viruses or bacteria from the nipple/areola prior to collection and this can alter the composition of its bioactive components. Hence, there are existing recommendations for hygienic practices around breastmilk expression, which include nipple/areola cleaning prior to collection and analysis [82,83].

Breastmilk samples should be collected in sterilised containers and immediately chilled at 4 °C to prevent bacterial growth and deterioration of breastmilk constituents [84]. Bacterial contamination can lead to an increase in protein content and a decrease in sugar content since bacteria utilise sugar as an energy source and can produce enzymes that cause proteolysis to breastmilk proteins, increasing the peptide fragments and hence increasing the risk of a false total protein content quantification. This can be addressed by adding protease inhibitors during sample collection and processing [85,86,87]. If breastmilk samples will not be analysed immediately, then they can be stored in −20 °C or −70 °C freezers [70,71,88,89]. Minimising freeze–thaw cycles is particularly important to preserve protein integrity and ensure consistent results [71,90,91,92].

**Figure 2:**
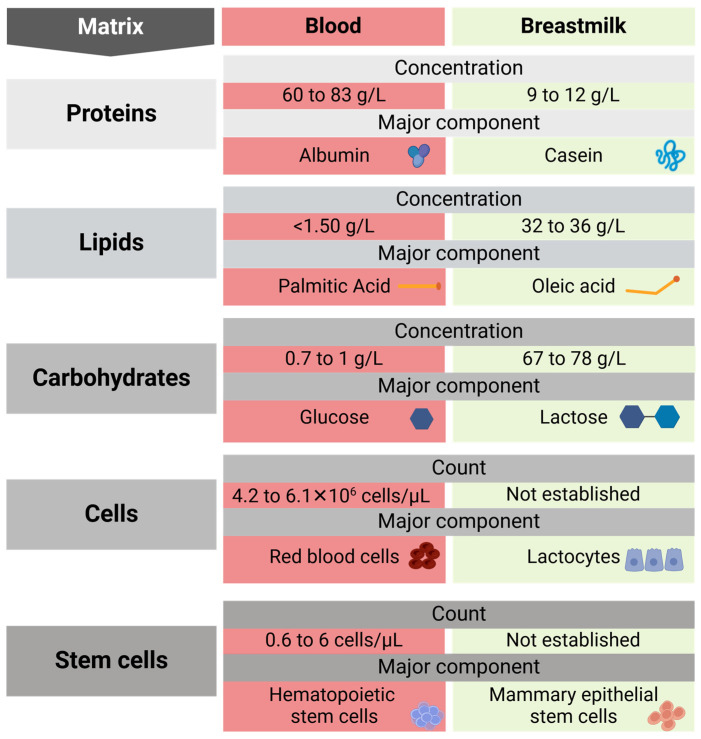
Comparison of the matrix present in blood and breastmilk [2,97,98,99,100,101,102,103].

A major analytical challenge in breastmilk quantification relates to the matrix, which is defined as the entire composition of the biological fluid, excluding the analyte of interest. The matrix of blood is different to the breastmilk matrix (Figure 2). For example, the breastmilk matrix includes a high abundance of casein protein, whereas albumin is the major protein in the blood. Lipid is more abundant in breastmilk compared to blood, with differences in the predominant type of lipid as well. The components of the biological matrix can significantly impact the precision and accuracy of quantification methods; this is known as the matrix effect [93,94]. For example, lipids in the breastmilk matrix can interfere with protein quantification. Even after lipid removal, residual lipids can compromise the accuracy and reliability of quantifying proteins. A better understanding of the breastmilk matrix will help to design methods that will minimise the matrix effect leading to better accuracy in quantification of bioactive components [95,96].

Addressing these pre-analytical and analytical challenges is essential for the quality and reproducibility of breastmilk analysis. In addition to these challenges, the different methods used for the detection and quantification of bioactive components in biological samples, such as mass spectrometry and immunoassays, each have distinct challenges and opportunities.

## 5. Mass Spectrometry

Mass spectrometry-based methods allow for quantitative analysis of the entire proteome [104,105,106,107] and can also be used in the sequencing of peptides and identification of post translational modifications [104,105,106,107]. Mass spectrometry is applicable across diverse research fields, including forensic toxicology, metabolomics, proteomics, pharma/biopharma, and clinical research [108]. It has been used to detect and quantify bioactive components such as proteins, lipids, HMOs, vitamins, and metabolites [109]. Mass spectrometry has advantages over other methods due to its sensitivity, accuracy, and ability to analyse complex biological matrices like breastmilk.

Mass spectrometry converts molecules present in a sample into ions using different ionisation techniques. The generated ions are then separated by mass analysers based on their mass-to-charge ratios (*m*/*z*), and detected according to their abundance [110,111]. Different types of analysers (e.g., time-of-flight, quadrupole) provide varying levels of resolution, speed, and sensitivity. The selection of an appropriate ionisation source [112,113] and mass analyser is critical, as it can maximise the capacity to identify signal molecules in breastmilk [108].

### 5.1. Challenges in Mass Spectrometry

Although mass spectrometry is regarded as ideal for the quantification of thousands of bioactive components, there are a number of challenges. Mass spectrometry can be influenced by the matrix of biological samples which can enhance or suppress the ionisation efficiency of the target analyte leading to under or overestimation of measured concentrations [93,94,114]. For instance, components in blood plasma such as phospholipids have been reported to decrease signals [115,116,117].

Another challenge associated with mass spectrometry analysis is the detection limit. Low molecular weight proteins such as cytokines can act as early biomarkers for many diseases [10,118]. However, these small molecules can be challenging to detect using mass spectrometry. In addition, low abundance signalling proteins such as hormones, may also be missed [119,120,121]. Breastmilk proteins can be divided into whey and casein fractions [2,122,123]. The most abundant proteins are casein (40% of total protein), α-lactalbumin, lactoferrin, secretory immunoglobulin IgA, lysozyme, and serum albumin [124]. These large proteins can hinder and mask the identification and detection of the smaller low-abundance proteins by suppressing the mass spectrometry signal resulting in inaccurate detection of potential biomarkers [125]. To enable the detection and quantification of low-abundance proteins, the high-abundance proteins can be removed prior to mass spectrometry analysis [96,121,125].

Finally, mass spectrometry techniques are expensive. The upfront cost of mass spectrometry equipment is higher compared to other equipment such as immunoassay instruments. The manual sample processing step is labor-intensive, which may lead to increased analytical imprecision. This can be improved by introducing automated sample processing platforms that facilitate the analysis and decrease human errors. Also, the need for technically trained personnel for operation and maintenance increases the overall expense, making the equipment less accessible.

### 5.2. Opportunities to Improve Mass Spectrometry

The challenges in using mass spectrometry can be overcome through the refinement of techniques to best suit the biological fluid and analyte of interest. Tailored protocols for sample preparation should be designed and implemented prior to analysis to address issues associated with the matrix effect. Simplifying sample preparation will decrease the time needed for method development and expedite sample analysis. In an attempt to simplify sample preparation steps, Dams et al. reported that reduced sample preparation time of biological samples prior to liquid chromatography–tandem mass spectrometry analysis affected the accuracy of the quantitative analysis due to the presence of endogenous matrix effects which were detected by mass spectrometry [94]. Breastmilk has a different matrix than blood and urine, so analytical methods that work for those matrices may not be suitable for breastmilk.

To overcome these issues, liquid chromatography–tandem mass spectrometry can be utilised. Although this approach may require more time and resources, it serves as a valid option in peptidomics due to its specificity, sensitivity, and ability to provide rigorous, accurate, and reliable results. Furthermore, liquid chromatography–tandem mass spectrometry enables the simultaneous measurement of multiple analytes [108,126].

Another opportunity to optimise mass spectrometry for breastmilk analysis is using bottom-up protein assay methods, which involves digesting proteins into smaller peptide fragments for quantification. This approach offers advantages such as high throughput, making it suitable for large-scale studies [96,127].

As mentioned above, proteins present at very low concentrations in breastmilk are harder to detect as they can be overshadowed by the highly abundant proteins. Introducing additional steps in the pre-analytical stage can remove the highly abundant proteins which facilitates the detection of low abundant proteins by mass spectrometry. This includes, but is not limited to, using organic solvents to remove large abundant proteins such as casein [128]. Casein can also be removed by acid-induced [129] or enzyme-induced precipitation [130], centrifugation [131,132], or by EDTA-induced dissociation [133]. In addition, commercially available protein depletion kits are used to remove highly abundant proteins [134,135,136].

Recent advances in the field of quantitative proteomics use multiple-reaction monitoring (also known as MRM) for biomarker validation. Multiple-reaction monitoring on a triple quadruple mass spectrometer provides superior sensitivity and selectivity for targeted peptides in a complex sample. Multiple-reaction monitoring also offers high precision in quantitation and a fast scan speed, which makes it a useful technology for validating biomarkers in a quick and robust manner [137,138]. Moreover, suitable controls, or even spike-in controls could be used to validate the quantified proteins [139,140]. Another new approach is the absolute quantification (AQUA) strategy, which is a mass spectrometry-based approach for the absolute quantification of proteins and peptides [141,142]. The absolute quantification is a crucial requirement for biomarker validation. In this technique, samples are spiked with known concentrations of isotopically labelled synthetic peptides [143]. Since these peptides are identical to their endogenous counterparts with respect to retention time, ionisation efficiency and fragmentation mechanism, they can act as an internal standard and serve as templates for method development to quantify both labelled and native analytes [141].

## 6. Immunoassays

Enzyme-linked immunosorbent assays (ELISA) are a rapid and convenient method for the detection of proteins in biological samples. It works by using monoclonal and polyclonal antibodies and enzymes to capture and detect individual proteins in a sample [144]. After binding to the target molecule, the sample is rinsed to remove any unbound protein, and then a secondary antibody labelled with an enzyme is added which produces a colour change [145,146].

Similar to ELISA, multiplex assays rely on antibodies to specifically capture and detect the analytes such as cytokines present in the sample. However, multiplex assays capture targets onto spherical beads in suspension while ELISAs generally rely on flat surfaces in multi-well plates to capture targets. The reporter system is based on fluorescence rather than the amplification of a colorimetric substrate as in ELISA [147]. Multiplex assays are designed to simultaneously detect multiple analytes at once by using different labelled probes or beads that each correspond to a specific target [10,148,149,150].

These immunoassay methods were largely developed and validated for use in blood and/or urine [3], with different matrix components compared to breastmilk. This poses a considerable challenge in the use of these existing methods for the analysis of breastmilk bioactive components and necessitates further protocol development and validation.

### 6.1. Challenges in Immunoassays

One of the unavoidable drawbacks of the commercially available ELISAs is the cross-reactivity with high molecular weight peptides [151,152,153]. In addition, they can also react with degraded forms of the bioactive peptides, limiting their specificity [152]. Moreover, ELISAs suffer from a lack of standardisation, which makes it difficult to compare results obtained from different assays [154,155,156,157]. ELISAs are time-consuming, as complex protocols and data analysis are performed manually, making it labor-intensive. In addition, it has limited throughput as it can only detect one analyte at a time. Finally, ELISAs have narrow dynamic ranges limited to a few orders of magnitude, making the quantification of analytes with varying concentrations challenging.

Challenges with multiplex assays include cross-reactivity with non-target proteins due to the increased targets present, potentially leading to incorrect quantification. Although a number of studies have used multiplex assays for the identification and quantification of proteins in breastmilk, the suitability is unclear, since these assays were designed and exclusively validated for plasma [158,159]. Moreover, as the number of targets increases, the risk of unspecificity of multiplex assays increases, leading to decreased accuracy for each analyte.

### 6.2. Opportunities to Improve Immunoassays

To address the challenges associated with quantifying breastmilk components, it is important to adapt methods specifically for use in breastmilk analysis. Optimal quantification methods need to be considered in terms of accuracy, sensitivity and robustness.

Cross-method validation can assist with investigating the accuracy of different quantification approaches (ELISA, mass spectrometry and multiplex). Through comparison of the same set of breastmilk samples, the accuracy of each of the methods can be assessed. To achieve this, spike-in controls need to be employed to determine recovery rates across all methods.

To determine the most sensitive method, the lowest concentration of the analyte that can be accurately detected needs to be assessed. Robustness refers to the ability of the method to provide consistent results under varying conditions. The method that will produce consistent and reliable results despite small variations in sample preparation and instrument settings can be considered the most robust method.

## 7. Quantification of Other Bioactive Components in Breastmilk

### 7.1. Lipids

Lipids form an integral part of breastmilk, being the second most abundant macronutrient after lactose and contributing more than 45–50% of the infant’s daily energy requirement [4,160,161]. Lipids found in breastmilk can be categorised into eight distinguishable subclasses known as fatty acids, glycerides, glycerophospholipids, sphingolipids, sterol lipids, prenol lipids, saccharolipids, and polyketides [162]. A challenge with quantifying lipids in breastmilk is their hydrophobic nature which necessitates the adoption of specific methods and protocols to separate and solubilise these lipids prior to analysis.

The three most commonly used methods for lipid extraction from breastmilk are the Modified Bligh and Dyer method, the Modified Folch method, and the Methyl tert-butyl ether method [163,164,165]. Ganeshalingam et al. recommend the Modified Bligh and Dyer method for breastmilk extraction because of its simplicity, superior lipid yield, and high throughput sample extraction [166]. In addition, there are other methods for the separation and quantification of breastmilk lipids such as gas chromatography and thin-layer chromatography [167,168,169,170].

Gas chromatography separates lipids based on their volatility and polarity with high sensitivity and specificity [171]. Some drawbacks associated with this method are that it requires sample derivatisation which can be time-consuming and it is not suitable for some lipid classes with low volatility such as phospholipids limiting its application [172].

Non-volatile compounds can be separated using thin-layer chromatography as this method separates lipids based on their polarity using a thin layer of adsorbent material, hence it is limited to qualitative identification of fatty acids [173]. This method is not commonly used in breastmilk lipidomics as it lacks the resolution of other mass-based approaches, and it cannot be used for quantitative purposes.

Hydrophilic interaction liquid chromatography techniques for lipid separation coupled with C30 reverse-phase chromatography can also be used for the analysis of lipids in breastmilk [166]. C30 reverse-phase chromatography allows the intra-class separation of breastmilk lipid isomers based on chain length and degree of unsaturation, making it the most suitable method for the identification of different classes of lipids when coupled with high-resolution tandem mass spectrometry [166].

Lipidomics is currently the most applied analytical method for lipid quantification and is based on different mass spectrometry techniques [174]. With current advances in instrumentation and techniques, there is potential for the development of improved separation of these complex lipids from the matrix and subsequent identification and quantification [167]. After separating the lipid layer by one of the previously mentioned methods, different approaches to lipidomics can be applied, such as shotgun lipidomics, which is commonly used for the characterisation of phospholipids. Another highly effective lipidomic approach is liquid chromatography–mass spectrometry which is used for the identification of triglycerides and phospholipids. This approach is based on a simplified analytical protocol which involves high chromatographic separation and a noticeable precision of detection leading to the identification of several lipids in a very short time [175].

### 7.2. Carbohydrates

Carbohydrates are the most prominent macronutrient in breastmilk [4]. Researchers have used many methods for quantifying lactose and other carbohydrates present in breastmilk. These methods can be categorised to enzymatic colorimetric (Dahlqvist), infrared spectroscopy, and liquid chromatography [5,176,177,178,179]. Infrared spectroscopy has been widely implemented in clinical settings despite its major drawback in distinguishing between lactose and oligosaccharides due to the similarity in their chemical structures [180,181].

McCune and colleagues recently assessed the influence of HMOs on measuring lactose levels in breastmilk, aiming to determine whether there are strong correlations between the lactose amounts quantified by different methods [180]. The assessed methods were based on enzymatic (megazyme enzymatic) and chromatographic (high-performance liquid chromatography with refractive index detection) assays. These two methods are currently validated for quantifying lactose in breastmilk without interference from HMOs [180].

### 7.3. Milk Analysers for Macronutrient Quantification

In the last few years, milk analyser technology has evolved to facilitate the rapid quantification of the total macronutrient content of breastmilk in fast-paced clinical settings such as in neonatal intensive care units and maternal and child health care institutions [182]. Milk analysers that incorporate infrared chromatography determine the quantity of the macronutrients by measuring their distinct infrared energy absorption wavelengths [84,183,184,185]. Other analysers are based on ultrasonic spectroscopy methods, in which high-frequency ultrasound radiation passes through the sample producing oscillations that alter the molecular arrangement of the sample. Macronutrient content is then quantified by measuring the transmission of the generated signals [186,187].

While both infrared and ultrasonic spectroscopy can be used to assess breastmilk macronutrient content, infrared spectroscopy is currently the more established and widely used method. Some studies have compared the accuracy and precision of these analysers versus the standard chemical methods [186,188]. These comparative studies demonstrated some differences between the values obtained from the milk analysers and the chemical methods. Results suggested that milk analysers require the application of conversion factors that improve accuracy [188,189]. A recent study applied machine learning methods to address the limitations of milk analysers in producing consistent results, suggesting some promising results [186].

## 8. Challenges and Opportunities in Quantifying Breastmilk Cells

In recent decades, researchers have identified various cell types derived from breastmilk. These cells have the potential to provide significant insights into the physiological functions of the mammary gland while also advancing therapeutic interventions for both mothers and infants. Breastmilk-derived cells may elucidate the factors contributing to lactation insufficiency and enhance understanding of the relationship between breastfeeding and breast cancer risk [64,190]. In addition, new approaches to stem cell transplantation and cell-based treatments for newborn diseases might be developed by employing comprehensive research on cellular components of the breastmilk [191].

Breastmilk-derived cells are considered to originate primarily from mammary glands, with a smaller contribution from peripheral blood [192]. These cells could be classified into immune cells such as lymphocytes and monocytes, and non-immune cells such as lactocytes, myoepithelial cells, progenitor cells, and stem cells [193]. Immune cells in breastmilk have been shown to perform a crucial role in protecting the lactating breast from infections, similar to their function in blood vessels and other tissues [194]. It is important to note that the immune cell population, notably macrophages, differs from those obtained from peripheral blood, indicating the distinctiveness of the immune cell population in breastmilk [195]. Breastmilk is also a rich source of multipotent mesenchymal stem cells due to their self-renewal and differentiation capabilities [196,197].

Animal studies have demonstrated that breastmilk cells can pass the digestive tract and translocate to different tissues such as the brain and lymph nodes [198,199]. It has been suggested that around 6% of the cell population in breastmilk consists of mesenchymal stem cells having multilineage potential [197,200]. Several investigations have employed single-cell RNA sequencing (scRNA-seq) of cells obtained from breastmilk in an effort to comprehend the maturation and sophistication of the mammary glands during lactation [190,201,202]. However, further research is needed to explore the application of breastmilk-derived cells in translational research for both mothers and infants [203].

Various techniques have been used to evaluate the diversity of cells obtained from breastmilk, such as cultivating them to determine their capacity for multilineage differentiation or assessing the expression of certain cell surface markers [200,204,205]. Recent findings indicate that transcriptome analysis of breastmilk-derived cells has become the preferred and most direct approach for investigating the dynamic cellularity of breastmilk [98,201,202,206,207].

However, studying breastmilk-derived cells presents challenges, as they cannot be extensively cultured over long passages, otherwise they would lose their in vivo phenotypes and may undergo dedifferentiation or phenotypic drift. Furthermore, the absence of a universally recognised protocol for the cultivation of breastmilk cells may contribute to differences in experimental results. For example, Fan and colleagues reported inconsistencies in the cellular composition of breastmilk compared to findings from a previous study [197,208]. Further research is required to overcome these obstacles and progress our understanding of the physiological significance of breastmilk cells.

## 9. Conclusions

Accurate quantification of bioactive components of breastmilk offers many opportunities for research on maternal and infant health and for biomarker discovery for different breast diseases. There are also many challenges linked to the quantification of these components and accurate quantification for human breastmilk requires consideration of the matrix effect and validation studies. Greater concordance between the results obtained from different methods will help to standardise the best method or approach to be implemented for the quantification of proteins, lipids, carbohydrates, and cells in breastmilk.

## Data Availability

No new data was created.

## Abbreviations

The following abbreviations are used in this manuscript:

AQUA	absolute quantification
ELISA	enzyme-linked immunosorbent
HMO	Human milk oligosaccharides
MRM	multiple-reaction monitoring
MS	mass Spectroscopy
